# Are movements of daytime and nighttime passerine migrants as different as day and night?

**DOI:** 10.1002/ece3.6704

**Published:** 2020-08-25

**Authors:** Bianca Michalik, Vera Brust, Ommo Hüppop

**Affiliations:** ^1^ Institute of Avian Research “Vogelwarte Helgoland” Wilhelmshaven Germany

**Keywords:** blackcap, diurnal versus nocturnal migration, dunnock, North Sea, radio‐telemetry, stopover

## Abstract

Even after decades of research, the migration of songbirds still holds numerous secrets. Distinct stopover and routing behavior of diurnally and nocturnally migrating songbirds has been stated in the 1960s, but empirical confirmation is yet lacking widely. We studied the behavior of individual diurnally migrating dunnocks and nocturnally migrating blackcaps by means of large‐scale automated radio‐telemetry. Birds were radio‐tagged during their stopover at the German North Sea coast. Our data indicate longer initial stopover duration in the diurnally migrating dunnocks, opposing the hypothesis of nocturnal migrants needing more time to recover due to their longer migratory flights. Nonetheless, dunnocks stopped over more often along their tracks as when compared to the nocturnally migrating blackcaps. Behavior *en route* did not differ as clearly between species challenging the general view of contrasting routings of diurnal and nocturnal migrants with regard to landscape and open water. Our results imply additional factors of relevance other than differences in species or daily migration timing per se. We discuss and highlight the need of detailed and individual based data to better understand stopover and routing behavior of songbirds in the environmental context.

## Graphical abstract

We use a large‐scale radio‐telemetry approach to directly compare the behaviour of dunnocks and blackcaps, a daytime and a nighttime migrant, on migration at the German North Sea coast. The diurnally travelling dunnocks stayed longer at stopover sites, while en route behaviour did not differ as clearly between species, challenging the general view of contrasting routings of diurnal and nocturnal migrants with regard to landscape and open water. Our results imply that detailed and individual‐based data are important to understand migratory strategies and properly judge risks and challenges individual species face on migration.

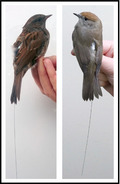

## 1 INTRODUCTION

The phenomenon of bird migration fascinates ornithologists since centuries. Large flock migrating birds such as the white stork (*Ciconia ciconia*) are attracting people, and their behavior during migration is getting better understood (e.g., Berthold et al., 2002; Shamoun‐Baranes et al., 2003). Through the advance of new tracking technologies, single birds can be followed during their whole annual cycle and even in remote areas (see Sokolov, 2011 for a review). There is, however, still astonishingly little known about the behavior of small migratory songbirds. They are particularly difficult to observe over larger distances as they are too small to carry high resolution tracking devices like GPS tags with remote data access (Bridge et al., 2011). Additionally, about two‐thirds of the European songbird species mainly or exclusively migrate at night (Berthold, 1993; Dorka, 1966; Martin, 1990). As songbirds only feed during daytime, nocturnal migration saves time for feeding (Alerstam, 2009; Kerlinger & Moore, 1989). Other benefits are improved physical flight conditions (weaker winds, reduced air turbulences, and lower surface temperature, Alerstam, 2011; Kerlinger & Moore, 1989; Shamoun‐Baranes, Liechti, & Vansteelant, 2017) and a higher relative humidity during the night reducing loss of water in flight (Alerstam, 2011; Kerlinger & Moore, 1989).

The migration of birds is subdivided into two main phases: stopovers used for energy intake and presumably recovery, and actual migratory flights (Alerstam, 2003). Due to the fact that the accumulation of fat storage takes comparably longer than burning these reserves during flight (Alerstam & Lindström, 1990), a greater proportion of time is spent at stopover sites than in sustained flight (Hedenström & Alerstam, 1997; Schmaljohann, Fox, & Bairlein, 2012; Wikelski et al., 2003). Although diurnally migrating birds can spot suitable stopover sites already in flight (Alerstam, 2009), nocturnal migrants need to extend their flights to dawn or at least need to adjust and fine tune nighttime choices of stopover sites after sunrise (Chernetsov, 2006). Nocturnal migrants cover larger distances at higher speeds and more often engage in longer flights, lasting up to the entire night (Liechti et al., 2018). Consequently, they might need more time to refuel and recover, resulting in extended times of stopover at single sites (Dorka, 1966). In contrast, diurnally migrating birds often restrict their migratory flights to daylight hours with improved flight conditions, that is, dawn and the early morning hours (Dorka, 1966). Diurnal migrants are thus more likely to cover shorter daily flight distances and accordingly stop and refuel more often along their track.

During flight, the availability of orientation cues differs between day and night. For example, guiding landscape structures are easier to detect during the day (Martin, 1990) which can be of particular relevance in prominent geographic areas such as at coastal environments. Consequently, diurnally migrating songbirds seem to follow coastlines readily and thereby avoid crossing open water (Drury & Keith, 1962; Hüppop et al., 2010). This holds especially true when the guiding structures do not deviate far from the overall migratory direction (e.g., Alerstam, 1990; Gruys‐Casimir, 1965; Van Dobben, 1953). Nocturnally migrating passerines, in contrast, seem to be less sensitive toward the overflown landscape, rather keeping their direction when encountering coastlines and continuing flights over the open water (Bruderer & Liechti, 1998; Diehl, Larkin, & Black, 2003; Eastwood, 1967; Lack, 1960, 1963; Myres, 1964). Still, nocturnal songbird migrants seem to avoid over sea flights more frequently toward the end of the night (Bruderer & Liechti, 1998; Fortin, Liechti, & Bruderer, 1999) and sometimes also follow coastlines (Brust, Michalik, & Hüppop, 2019; Buurma, 1995; Richardson, 1978). Some diurnal migrants regularly cross the open sea as well (Gruber & Nehls, 2003; Hüppop et al., 2010; Van Dobben, 1953). These partly contrasting findings are derived from observations at individual sites rather than from following individual birds. Accordingly, knowledge on the behavior of individual diurnal and nocturnal migrants regarding stopover ecology and routing is scarce. Furthermore, quantitative comparisons of the proportions of individuals of the same species deciding for different routes at the coast are lacking. A better understanding of individual behavior during migration is an important basis to better assess potential natural and anthropogenic risks the birds might encounter *en route*. When weather conditions deteriorate during flight over the open water, migrating land birds may face a severe risk of drowning (e.g., Diehl, Bates, Willard, & Gnoske, 2014). For birds attempting an “emergency landing” under these averse conditions, collision risk with artificial structures offshore may be particularly high (e.g., Aumüller, Boos, Freienstein, Hill, & Hill, 2011; Hüppop, Hüppop, Dierschke, & Hill, 2016; Newton & Little, 2009). Especially in the light of the worldwide growing offshore wind energy industry (Lee & Zhao, 2020) collisions pose a serious threat to birds migrating offshore (see Hüppop, Michalik, Bach, Hill, & Pelletier, 2019 for a recent review). Given that many populations of migratory songbird are rapidly declining in numbers (e.g., Bairlein, 2016; Berthold, Fiedler, Schlenker, & Querner, 1998), such knowledge has the potential to contribute to a necessary year round protection (Diehl, 2013; Hüppop, Ciach, et al., 2019; Runge et al., 2015) by uncovering risks during migration as an important and energetically highly demanding part of their life cycle.

**Figure 1:**
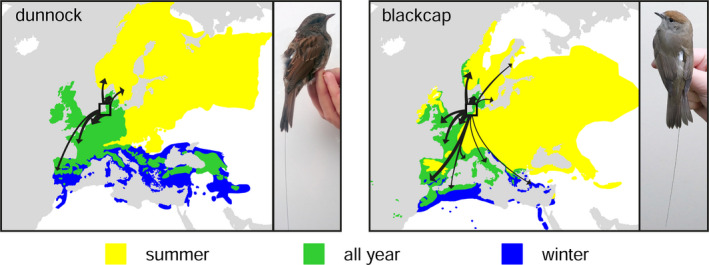
Species distribution maps of dunnocks (left) and blackcaps (right). Maps are shown in Mercator projection. Arrows represent hypothetical migration routes of individuals migrating through our study area (box) as derived from recoveries of birds ringed on Helgoland (Dierschke et al., 2011). Species distribution data were kindly provided by BirdLife International (2019). Note that a small fraction of blackcaps also migrates until sub‐Saharan Africa (not shown)

In our study, we compared the migration and stopover behavior of a diurnal and a nocturnal migrant at the German North Sea during autumn as well as during spring migration season. The two study species, dunnock (*Prunella modularis*, Dorka, 1966; Glutz von Blotzheim & Bauer, 1985, diurnal migrant) and blackcap (*Sylvia atricapilla*, Berthold, Gwinner, Klein, & Westrich, 1972; Glutz von Blotzheim & Bauer, 1991, nocturnal migrant), are both mainly insectivorous songbirds which include plant food in their diet during autumn migration and winter (Berthold, Querner, & Schlenker, 1990; Bishton, 1986). Throughout Germany, they co‐occur in the same habitats but differ in their use of microhabitat: Dunnocks usually forage on the ground or from small plants (Bishton, 1986; Glutz von Blotzheim & Bauer, 1985), whereas blackcaps usually search food in bushes and trees (Berthold et al., 1990; Glutz von Blotzheim & Bauer, 1991). Both species have comparably timed migration seasons with spring peak migration through our study area in dunnocks in late March/early April and in blackcaps in the end of April (Dierschke, Dierschke, Hüppop, Hüppop, & Jachmann, 2011). Autumn migration through our study area peaks in dunnocks in the end of September and in blackcaps from late September to mid‐October but ranges in both species until November (Dierschke et al., 2011). Birds of both species passing through our study area migrate in a south‐westerly direction from their breeding grounds in Germany, Denmark, and Southern Scandinavia toward their main wintering areas which range in both species from western to south‐western Europe and in blackcaps until northern Africa (Figure 1; Bairlein et al., 2014; Dierschke et al., 2011; Zang, 2001, 2005). Spring migration through our study area takes place in a north‐easterly direction (Figure 1; Bairlein et al., 2014; Dierschke et al., 2011; Zang, 2001, 2005). Both species do not forage in migratory flight and thus need to stop over for refueling. Concluding, the two species resemble each other in many ecological aspects. The major difference lies in their daily timing of migration. We thus chose these two species as representatives of each group of songbird migrants to study their behavioral differences during migration at the German Bight.

**Figure 2:**
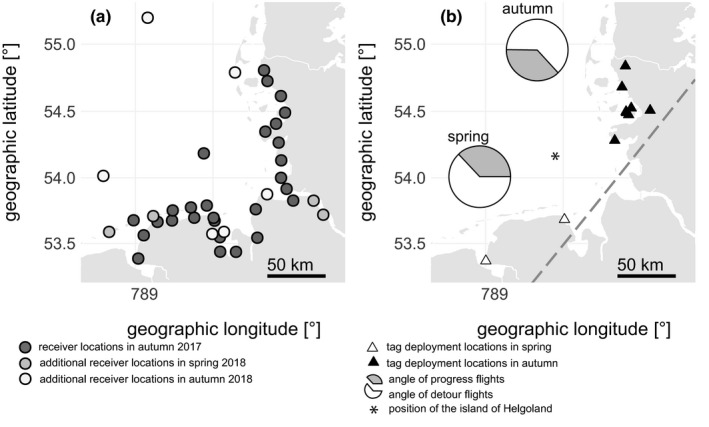
German Bight (south‐eastern North Sea) showing (a) locations of Motus network receivers and (b) locations of tag deployment and the island of Helgoland (asterisk) as well as progress/detour bearings during either migration season (pie charts) and threshold latitude/longitude for offshore/alongshore flight classification (dashed line) as described in the methods section

We used an array of automated radio receiving stations covering large parts of the south‐eastern North Sea, that is, the German Bight (Brust et al., 2019). Individuals of both species were captured and tagged during spring as well as during autumn migration at the same coastal stopover sites. Consecutively, we tracked their stopovers and flights along the German Bight. The diagonal distance across the German Bight is roughly 150 km (Figure 2). Direct crossing of the open water would take the birds about three and a half hours of nonstop flight in neutral winds (see Bruderer & Boldt, 2001 for species specific airspeeds). In our study area, the course of the coastline does not deviate too far from the species' overall direction of migration (Bairlein et al., 2014) and coastal migration seems to be more pronounced than offshore migration in this area (Hüppop et al., 2010; Hüppop, Dierschke, Exo, Frederich, & Hill, 2006). Nevertheless, large numbers of individuals of both species are ringed each migratory season at the offshore island of Helgoland (Dierschke et al., 2011) indicating a considerable amount of birds flying for at least 50 km offshore.

Assuming similar habitat choice during stopover, we expected our study species to stay for a similar amount of time and to cover similar overall distances in the German Bight area. Still, we expected the diurnally migrating dunnocks to move over shorter distances per day and to stop over repeatedly for rather short time periods. In addition, we expected their course to be more influenced by the coastline. The night migrating blackcaps, in contrast, were expected to cover larger flight distances at once and to stop over less often but for longer time spans. We expected their course to be less affected by the coastline.

## 2 MATERIAL AND METHODS

### 2.1 Field methods and telemetry system

Over two consecutive years, we mist‐netted 79 dunnocks and 72 blackcaps. In autumn, 46 dunnocks and 40 blackcaps were caught between 27 September and 24 October 2017 and between 3 and 14 October 2018. In spring, each species was caught during its respective peak of migration (Dierschke et al., 2011), that is, 33 dunnocks between 20 and 27 March and 32 blackcaps between 8 and 21 April 2018. Both species were caught at the same coastal stopover sites in Schleswig‐Holstein, Germany, during autumn and in Lower Saxony, Germany, during spring, respectively (Figure 2b). Each bird was equipped with an individually coded nanotransmitter (type NTQB, Lotek Wireless Inc.) using a Rappole‐type leg loop harness adjusted to body size (Rappole & Tipton, 1991). In addition, the bird was ringed and its fat score was estimated visually according to the Kaiser (1993) scale prior to its release. Radio signals of the transmitters were automatically recorded by an array of receiver stations (Figure 2a, Francis, Taylor, & Crysler, 2016; Taylor et al., 2017) covering the coastline and several islands along the German Bight. To create a map of the German Bight, ESRI shape files of the coastlines of Europe were taken from the world vector shoreline dataset of the GSHHG database (Wessel & Smith, 1996), which is available at the NOAA National Centers for Environmental Information website (https://shoreline.noaa.gov).

### 2.2 Telemetry data preparation

Raw receiver data were filtered automatically for individual tag signals by motus.org (Crewe et al. 2018) and downloaded via the Motus R package (Brzustowski & Lepage, 2018). We subsequently applied an additional data evaluation routine as described in detail in Brust et al. (2019) to exclude possible false‐positive detections from our data (please see Crewe et al. 2018, chapter 5 for types of error in Motus data). We chose a threshold probability estimate of being a false positive of 0.67. We decided to use this rather conservative threshold in order to be sure to eliminate most of the false‐positive detections that occur at some of our receiver sites and may arise, for example, from sources like marine radio, amateur radio, or mobile communication traffic (Crewe, Crysler, & Taylor, 2018). Our additional data evaluation resulted in 133,422 detections. We also included recaptures of individuals at the tag deployment sites during the field seasons in the analysis (7 data points of 4 individuals).

We combined all detections of each bird into an individual “track.” Sunrise and sunset times for the time stamps of the start of each recording were calculated using the R package RAtmosphere (Biavati, 2014). We identified the first receiver detecting the bird within a 20 km range of its tag deployment site. All signals recorded by this receiver and others nearby, that is, a maximum of 2.6 km apart, were included to calculate an “initial stopover duration,” until the bird was detected elsewhere for the first time. This approach includes landscape movements into the estimation of stopover duration which differs from classical measurements that are usually based on recaptures or observations at the same spots (see Kaiser, 1999 for a review). Our wider approach of defining the stopover duration enables us to better compare the species' behavior in their migratory context including their different choice of microhabitat.

For the remaining detections of each track, we distinguished between three behavioral categories in the style of Smetzer, King, and Taylor (2017). For category differentiation, we defined threshold values from histograms of recording duration, distance between subsequently recording receivers, and estimated ground speeds between subsequent recordings. “Along track stopover” either comprised detections at the same receiver station for more than one hour or was indicated by slow movements (< 5 m/s, Smetzer et al., 2017) with subsequent detections within three days at ranges of less than 32 km. “Flight” comprised movements at reasonable rates, that is, flight speeds of 5–26 m/s over ground (see Bruderer & Boldt, 2001 for species specific airspeeds), as well as simultaneous detections, that is, detections with very fast estimated flight speeds of >26 m/s between two receiver sites, and recordings for less than 35 min at only a single receiver station. All other detections at slow speeds (<5 m/s) were defined as “unknown” which reflect discontinuous recordings indicating gaps in space and/or time.

From these data, we calculated the “duration of along track stopovers” in days for each individual as well as the proportion of individuals taking at least one additional stop along their track per species and season, respectively. In addition, we calculated for each individual the “time spent at the German Bight” as the number of days between tag deployment and last recording. A “daily mean flight speed” for each individual was calculated as the summed up distance between receivers recording “flights” or slow stopover movements divided by the duration of the respective recordings in whole days.

### 2.3 Routing: “Detours”

In order to look at “detours” taken by each individual, we combined all detections which represent changes of an individual between receiver sites but were not simultaneous detections. From this dataset, we analyzed the headings from one receiver to the next. We classified headings between 315° and 90° in spring and between 135° and 270° in autumn as “progress tracks” (Figure 2b), which were movements in the species’ overall direction of migration as retrieved from ringing recoveries (Bairlein et al., 2014; Dierschke et al., 2011; Zang, 2001, 2005). All other headings including reverse migration were classified as “detour tracks”. For each individual, we calculated the cumulative length of their respective “progress” and “detour tracks” as well as the proportions of individuals taking detours per species and season.

### 2.4 Routing: Crossing or coasting

For 48 individuals, we extracted “sustained flights.” These were defined as continuous recordings by either> 3 receivers, which had to be located> 2.6 km apart from each other, or by two receivers being at least 35 km apart from each other, with time gaps between subsequent detections of < 7 hr. To avoid multiple sampling, we included only the first “sustained flight” of each individual into further analyses. The resulting 48 “sustained flights” were classified into offshore or alongshore oriented flights (Table 2). Offshore flights either included recordings at the island of Helgoland (Figure 2b) or met the following criteria: The latitude of the last recording receiver above 54° was above 54.135°, and the first longitude of recording receivers at latitudes below 54° was below 8.08° in autumn or vice versa in spring (Figure 2b). All other options of “sustained flight” were classified as alongshore. For more details on the procedure, see Brust et al. (2019).

### 2.5 Statistics

All statistics were performed using R 3.5.2 (R Core Team, 2018). Since the data of stopover behavior and recording features as explained above were not normally distributed, we calculated median values and their 25% and 75% quantiles. Comparisons of these values between species were performed separately for spring and autumn, using the nonparametric Kruskal–Wallis test. Proportions of count data were compared between species and seasons using the binomial test.

### 2.6 Ethical note

Experiments were approved by the Ministry of Energy Transition, Agriculture, Environment, Nature and Digitalization (MELUND) for birds caught in Schleswig‐Holstein, license number V244‐69134/2016(92‐8/16), and by the Lower Saxony State Office for Consumer Protection and Food Safety (LAVES in Lower Saxony, license number 33.19–42502–04‐16/2349), respectively.

## 3 RESULTS

### 3.1 Daily timing of migration

**Figure 3:**
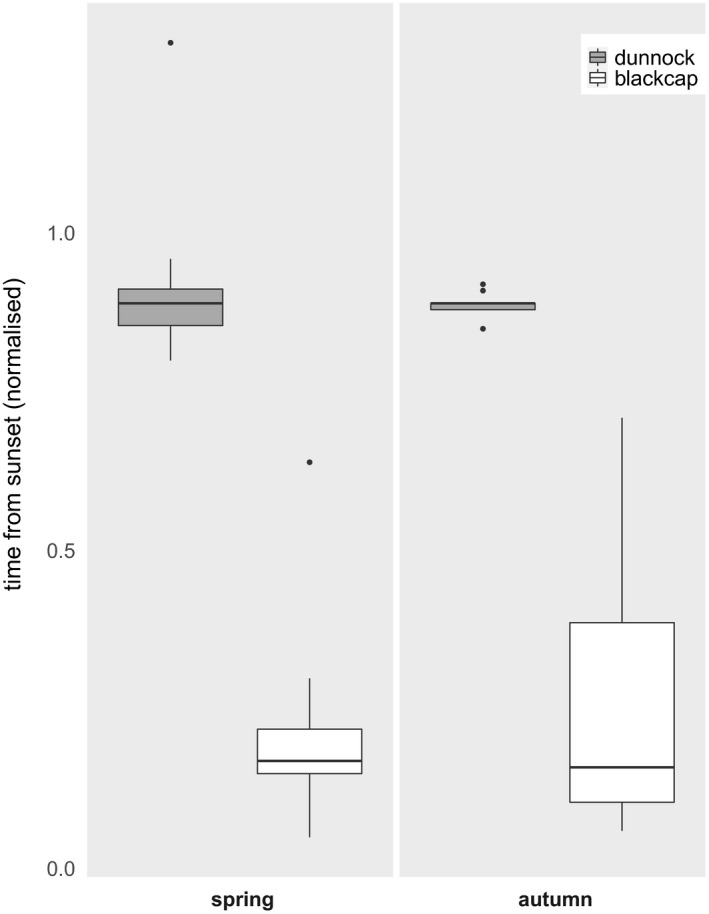
Tukey style box and whisker plot of daily start times of sustained flights of dunnocks (gray boxes, *n* = 29) and blackcaps (white boxes, *n* = 19) recorded at the German Bight during spring and autumn (corrected for night length). Zero refers to sunset, one to sunrise

**Figure 4:**
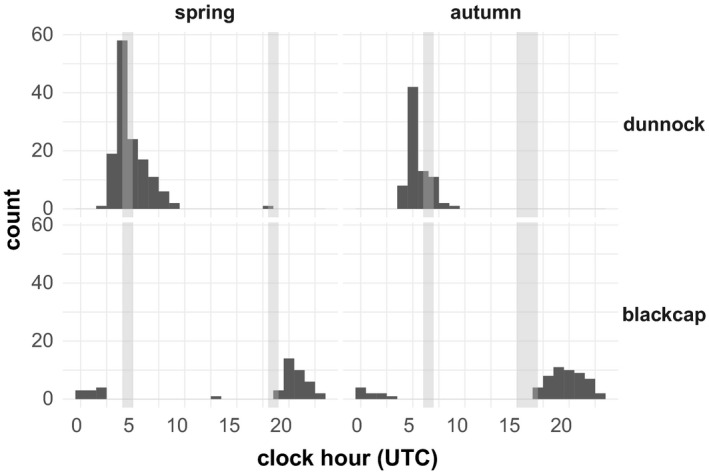
Hours (UTC) of flights recorded per species and season. Gray bars indicate times of sunrise and sunset during each season

From the 151 tagged birds, 123 individuals have in total been detected by the automated receiver network during spring (32 dunnocks, 28 blackcaps) and autumn migration (33 dunnocks, 30 blackcaps). “Sustained flights” were tracked in 48 individuals (29 dunnocks, 19 blackcaps). “Sustained flights” of dunnocks started in the early morning, mainly before sunrise, while those of blackcaps predominantly started in the first quarter of the night (Kruskal–Wallis test with multiple comparisons, χ^2^ = 34.06, *p* < .001, Figure 3). We recorded “flight” behavior in dunnocks starting up to three hours before sunrise and lasting up to five hours into the day (Figure 4). Blackcaps, in contrast, fully restricted “flights” to the night, avoiding even the hours of morning twilight (Figure 4). Exceptions from this general pattern were only recorded in spring. One out of the 16 “sustained flights” of dunnocks recorded in spring took place around sunset and one out of nine “sustained flights” of blackcaps recorded in spring took place during midday. Similar exceptions were not recorded in autumn.

### 3.2 Stopover behavior

**Figure 5:**
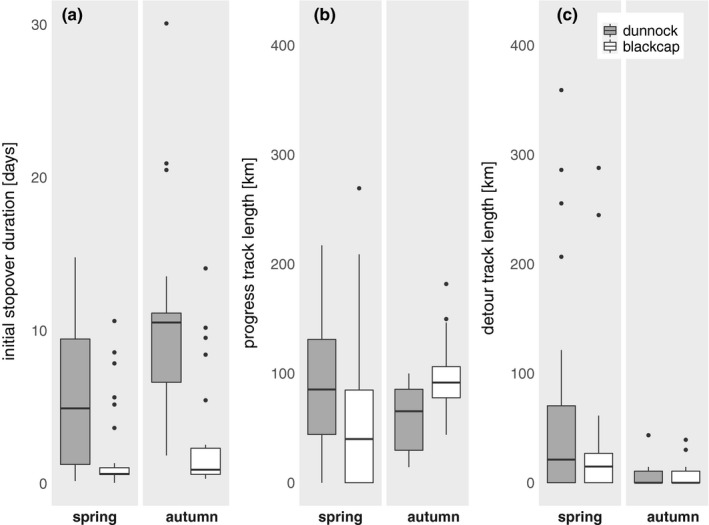
Tukey style box and whisker plot of a) initial stopover duration, b) progress track length, and c) detour track length at the German Bight in dunnocks (gray boxes) and blackcaps (white boxes) during spring and autumn migration season. See Table 1 for statistics

A minimum “initial stopover duration” in close proximity to the location of tag deployment could be determined in 55 dunnocks and 50 blackcaps (Table 1). Fat scores of these individuals estimated at the time of tag deployment differed only slightly between species and ranged in both species around a value of at least 3 (dunnock: 3.00 ± 0.55 (median ± mean deviation from median), blackcap: 3.75 ± 0.93). Initial stopovers were recorded equally likely in both species and seasons (spring: dunnock: 27 of 32, blackcap: 28 of 28, binomial test, χ^2^ = 2.95, *p* = .086; autumn: dunnock: 28 of 33, blackcap: 22 of 30, binomial test, χ^2^ = 0.67, *p* = .414), but dunnocks stopped over significantly longer than blackcaps at their respective tag deployment sites (Table 1, Figure 5 a). In spring, a higher proportion of dunnocks was found to have at least one additional stopover “along the track” (dunnock: 19 of 32, blackcap: 4 of 28, binomial test, χ^2^ = 11.01, *p* < .001). In autumn, both species were equally likely to stop over “along the track” (dunnock: 10 of 33, blackcap: 7 of 30, binomial test, χ^2^ = 0.11, *p* = .735). Duration of “along track stopovers” was quite variable in individuals but comparable between species (Table 1). Taking stopovers and movements into account, dunnocks stayed longer than blackcaps in the German Bight area in both seasons (Table 1).

**Table 1:** Track characteristics of dunnocks and blackcaps in spring and autumn calculated from radio‐telemetry data at the German Bight. Shown are median values of the data with their 25% and 75% quantiles and Kruskal–Wallis statistics with significant tests in bold.

Variable	Season	Dunnock				Blackcap				Kruskal–Wallis test	
		Median	25%	75%	*n*	Median	25%	75%	*n*	χ^2^	*p*
Initial stopover duration [days]	Spring	4.9	1.2	9.4	27	0.6	0.6	1.0	28	9.92	**.002**
	Autumn	10.5	6.6	11.1	28	0.9	0.6	2.3	22	22.74	**<.001**
Along track stopover duration [days]	Spring	6.9	1.8	11.6	19	1.4	0.8	7.4	4	0.66	.420
	Autumn	0.2	0.1	3.0	10	0.1	0	1.0	7	0.61	.437
Time spent at the German Bight [days]	Spring	15.0	12.0	18.2	32	2.0	1.0	7.5	28	27.94	**<.001**
	Autumn	12.0	10.0	15.0	33	8.5	2.2	11.0	30	11.01	**<.001**
Progress track length [km]	Spring	87.2	64.1	136.4	24	68.9	53.9	117.8	11	0.85	.356
	Autumn	65.4	29.7	85.5	17	91.6	77.7	106.2	17	8.99	**.003**
Detour track length [km]	Spring	59.6	17.5	106.4	20	26.9	17.6	54.1	10	1.12	.291
	Autumn	14.5	10.6	14.5	5	14.5	14.2	30.1	5	0.10	.748
Mean speed [km/day]	Spring	32.3	14.7	44.9	24	21.9	6.2	41.8	22	0.39	.533
	Autumn	32.6	22.3	79.6	24	50.8	15.0	77.7	17	0.13	.724

### 3.3 Routing behavior

Once the birds left their stopover sites and embarked on flights, dunnocks and blackcaps could be followed over similar distances of “progress tracks” and “detours” in spring (Figure 5b,c, Table 1). In autumn, “progress tracks” were longer in blackcaps than in dunnocks, while “detours” remained comparable in length (Figure 5b,c, Table 1). Comparing the seasons, “detours” were longer in spring than in autumn (Table 1, Kruskal–Wallis test, dunnock: χ^2^ = 12.12, *p* < .001, blackcap: χ^2^ = 4.27, *p* = .039). The “progress track” lengths differed between seasons in both species but in opposite tendencies (Table 1, dunnock: Kruskal–Wallis test, χ^2^ = 3.00, *p* = .083, blackcap: Kruskal–Wallis test, χ^2^ = 5.28, *p* = .022). The proportion of dunnocks taking “detours” was higher in spring (20 of 28) than in autumn (5 of 17, binomial test, χ^2^ = 5.96, *p* = .015). In blackcaps, there was no difference in the proportions of birds taking “detours” between seasons (spring: 10 of 17, autumn: 5 of 17, binomial test, χ^2^ = 1.91, *p* = .167).

In total, 9 of 29 “sustained flights” in dunnocks and 6 of 19 in blackcaps were oriented offshore. The species generally did not differ in their respective proportions of “sustained flights” along the coastline or offshore (Table 2). The proportion of dunnocks that took an offshore oriented “sustained flight” in spring was not higher than in autumn (Table 2). In blackcaps, accordingly, proportions of offshore oriented “sustained flights” did not differ between spring and autumn (Table 2).

**Table 2:** Observed proportions and corresponding binomial statistics of sustained flights occurring along‐ and offshore at the German Bight per species and season

Species	Spring		Autumn		Binomial test	
	Alongshore	Offshore	Alongshore	Offshore	χ^2^	*p*
Dunnock	9	7	11	2	1.53	.216
Blackcap	6	3	7	3	<0.01	1
Binomial test	χ^2^ < 0.01	*p* = 1				

## 4 DISCUSSION

Tagged dunnocks and blackcaps showed clearly distinct daily flight schedules at the German Bight, confirming their assignment as primarily diurnal and nocturnal migrants, respectively. Contrasting our predictions, we found the diurnally migrating dunnocks to stay longer in our study area and to stop over longer in comparison to the nocturnally migrating blackcaps. This finding challenges the general assumption that nocturnal migrants need more time to recover than diurnal migrants due to their longer daily migratory flights. In line with our predictions, at least in spring, dunnocks stopped over more often along their track. But we did not find clear hints that they might have been more tempted to follow the coastline than blackcaps. Both tracked detours and proportions of tracked offshore flights did not differ between the species. Our findings question the general view of contrasting routing behavior of diurnal and nocturnal migrants with regard to guiding effects of coastlines and open water.

### 4.1 Daily timing of migration

Our radio‐telemetry data nicely confirmed the different daily migration times of dunnocks and blackcaps. Flights of dunnocks were mainly recorded in the early morning hours but began even up to three hours before sunrise. An early onset of up to one hour before sunrise has been found repeatedly in dunnocks (summarized in Glutz von Blotzheim & Bauer, 1985). Diurnal migrants, in general, are supposed to begin flights very early in the day, usually during the first dim light of dawn (Alerstam, 1990; Bruderer, 1999; Dorka, 1966). The hours close to sunset, in contrast, are less often but nonetheless regularly used for migratory flights in diurnal migrants (Alerstam, 1990) and dunnocks in particular (Glutz von Blotzheim & Bauer, 1985), which we also found in our data. Nocturnal migrations have been reported, too, for example, in dunnocks migrating offshore (Drost, 1960; Hill, Debus, Rebke, & Weiner, 2014; Kulik, Skov, Hill, & Piper, 2020; Stahl & Nehls, 2004). Despite we also observed occasional nocturnal activity in our tagged dunnocks (data not shown), we did not record strictly nocturnal flights. In contrast to diurnal migrants, nocturnal migrants are usually setting on flights about half an hour after sunset (Alerstam, 1990; Müller et al., 2016), a behavior that is also well reflected in our tagged blackcaps. Still, shorter movements in order to find suitable feeding patches or resting sites during the day are not unusual (Alerstam, 2009; Chernetsov, 2006). In line with this, we also found a flight of one tagged blackcap during midday. All our tagged blackcaps terminated their flights well before dawn. This seems surprising considering anecdotal observations of blackcaps arriving en masse to roost in bushes during the morning (Schmid & Grossmann, 1988). Nocturnally migrating birds regularly extend their flights into the early morning, especially when crossing larger ecological barriers (e.g., Archibald, Buler, Smolinsky, & Smith, 2016; Bourne, 1980; Bruderer & Liechti, 1998; Diehl et al., 2003; Hüppop et al., 2010; Myres, 1964). Ellegren (1993), however, calculated from ringing recoveries in the Baltic area that migratory flights of nocturnal migrants took place during 20 ‒ 40% of the dark period. In line with this, Bolshakov, Bulyuk, and Chernetsov (2003) reported from the Courish Spit in the eastern Baltic that most nocturnally migrating passerines ended their flights at dawn at about 90 ‒ 40 min before sunrise and landings after sunrise have only been observed very rarely. Given the relatively short geographic range of our receiver network (Figure 2 a), we might have predominantly recorded shorter migratory flights occurring earlier in the night as suggested by Ellegren (1993).

### 4.2 Stopover behavior

The initial stopover behavior of our tagged dunnocks and blackcaps contradicts our expectations: blackcaps left the area of tag deployment faster than dunnocks. We based our prediction of nocturnally migrating blackcaps stopping over for longer time than diurnally migrating dunnocks on their respective need to accumulate more fat in order to undertake longer flights as suggested by Dorka (1966). In our study, the fat scores of both species indicated that they had enough energy stored to resume migration without a refueling delay (Langslow, 1976). It is hence likely that the birds in our study did not stop over at our coastal tag deployment sites because of a shortage of fuel. Encountering a large water body, for instance, is known to favor termination of flights also in nocturnal migrants (Bruderer & Liechti, 1998; Jenni & Schaub, 2003). Especially when dawn proceeds, birds migrating in coastal environments have been observed regularly to reorient toward land to avoid the risky crossing of open water (Archibald et al., 2016; Bourne, 1980; Bruderer & Liechti, 1998; Diehl et al., 2003; Myres, 1964; Nilsson & Sjöberg, 2016). The maximum distance a bird needs to cross the water body in our study area is with some 150 km relatively short (Figure 2), and direct crossing of the open water would take birds of our study species about three and a half hours in neutral winds (see Bruderer & Boldt, 2001 for species specific airspeeds). Still, with the increasing number of offshore wind farms in the German North Sea (Lee & Zhao, 2020) the passage becomes increasingly risky. Many mass mortality events of migrating land birds, including our study species, have been reported from artificial offshore structures in the North Sea (Aumüller et al., 2011; Hüppop et al., 2016; Hüppop, Michalik, et al., 2019).

The birds in our study might have terminated their flights due to the confrontation with the open water. In this case, the duration of stopover should be relatively short, reflecting only the time needed to rest and recover. Most of our tagged blackcaps indeed left their coastal stopover sites during the next night in either season. Dunnocks, in contrast, stopped over at the tag deployment sites for a more variable amount of time, usually a few days indicating additional factors of relevance for stopover decisions (see Müller et al., 2016 for a recent review).

Following the birds further along their tracks, we found the expected difference in stopover ecology, at least in spring: A higher proportion of dunnocks stopped over repeatedly as compared to blackcaps. Interestingly, this difference was not present in autumn. Our data support the hypothesis that the diurnally migrating dunnocks are more likely to stop over repeatedly, at least in spring, as when compared to the nocturnally migrating blackcaps (Dorka, 1966). This hypothesis is further supported by the fact that dunnocks spent more time in the detection range of our receiver network than blackcaps in both seasons. The proportions of individuals having at least one additional stop along their tracks are, however, quite low in both species, which might reveal limitations of our recording design. The array of receivers covers only a small part of the species' overall migration route (Figure 1; Bairlein et al., 2014; Dierschke et al., 2011; Zang, 2001, 2005). Only including actually recorded stopovers in the data set, we may have underestimated along track stopovers in both species. This is further indicated by the discrepancy between the relatively short time of recorded stopovers and the total recording time which the birds spent in the area of the German Bight (Table 1). Our recording design was, however, the same for both species. Bearing these considerations in mind, we are confident that our data point to differences in along track stopover behavior between the diurnally migrating dunnocks and the nocturnally migrating blackcaps within our study area.

### 4.3 Routing behavior

Our telemetry data on routing behavior of the tagged dunnocks and blackcaps are not as clear and straightforward to interpret as expected. They neither support nor speak thoroughly against our hypothesis that the course of the diurnally migrating dunnocks should be more distracted by a misleading coastline (Drury & Keith, 1962; Hüppop et al., 2010; Hüppop, Michalik, et al., 2019). The lengths of detour tracks were comparable between the two study species in both seasons. Still, the tagged dunnocks were more likely to take detours and stayed longer in the area of the German Bight in *spring* than the tagged blackcaps. Particularly, young birds that successfully followed the coastline in their previous autumn might undertake time‐consuming flights exploring the coast in spring, as, for example, documented in young blackpoll warblers at the Gulf of Maine (Brown & Taylor, 2015).

Alternatively, one might speculate that the dunnocks tagged in spring might have been less time pressed and might have had additional time to explore the area. We tagged the dunnocks about two weeks earlier during spring than the blackcaps which might support this suggestion. We do not think, however, that this could be an explanation for our findings since, in both species, we tagged the birds only on good migration days when we caught many individuals of the same species. We therefore assumed the tagged birds to be still in migratory mood when stopping over at the tag deployment site. Although breeding times largely overlap in the two species, dunnocks migrate (Dierschke et al., 2011) and also start breeding slightly earlier than blackcaps (Glutz von Blotzheim & Bauer, 1985, 1991), which rather speaks against less time constraints in dunnocks. The breeding destinations of the individuals we tagged in spring were, however, not known. Still, both study species breed at our site of spring tag deployment in very low numbers (pers. obs.). Furthermore, breeding densities of both species are low as well in the coastal areas of Lower Saxony (Krüger, Ludwig, Pfützke, & Zang, 2014). We were hence confident that the vast majority of our birds tagged in spring did not breed in close proximity to our receiver stations in Lower Saxony. But we could not be sure about the migratory distances yet to cope for these birds which might partly explain the observed longer recording duration of dunnocks tagged in spring.

We think, however, that the higher proportion of dunnocks observed to take detours in spring could hint to another phenomenon, so‐called landscape movements (see Schmaljohann & Eikenaar, 2017 for a recent review). As revealed from recent radio‐tracking studies, songbird migrants might sometimes leave a stopover site to search for another stopover site nearby (Mills, Thurber, Mackenzie, & Taylor, 2011; Stach, Fransson, Jakobsson, & Kullberg, 2015; Taylor et al., 2011) or might perform short exploratory flights, for example, to check wind conditions aloft (Schmaljohann et al., 2011).

Remarkably, we found in both species detour tracks to be longer in spring than in autumn. This contrasts the general notion that, in many species, spring migration should be more goal‐directed and thus faster than autumn migration (e.g., Berthold, 1990), as the birds should be pressed for a timely arrival at their breeding grounds in order to compete for high quality territories and mates (Kokko, 1999). A few studies indicate indeed faster spring migration (e.g., Cochran, 1987; Nilsson, Klaassen, & Alerstam, 2013; Schmaljohann, 2018; Yohannes, Biebach, Nikolaus, & Pearson, 2009) and, for example, blackcaps migrating through Europe have been found to cover their migratory distance about 60% faster in spring than in autumn (Fransson, 1995). We can only speculate why our data seemingly contrast these findings. We also have to bear in mind that dimensions of the migratory routes which were covered by our receiver array might have differed between the seasons making conclusions difficult. As a consequence, in our study, comparisons *between seasons* have to be regarded with caution.

Interestingly, blackcaps could be followed over longer progress tracks in *autumn* as when compared to dunnocks. The wintering grounds of both species range from south‐western Europe to northern (Figure 1; Bairlein et al., 2014; Dierschke et al., 2011; Zang, 2001, 2005). We could thus be quite sure that the vast majority of birds we tagged at their coastal stopover sites during autumn were migrating through our study area. Since we tagged the individuals of both species at the same time and at the same spots during autumn migration, the recording conditions, that is, the radio‐receiver array and thus the detection probability, have been essentially the same for both species. Longer recorded progress tracks then suggest that the blackcaps tagged in autumn might have followed the coastline more thoroughly than the dunnocks. We therefore conclude that our radio‐telemetry data indicate differences in routing behavior of the two species at our study site in autumn.

There was, however, no species difference in proportions of tagged individuals embarking on offshore flights in neither season. This finding was rather unexpected following the general notion that nocturnally migrating birds being less sensitive toward the overflown landscape and to readily continue flights over the open water (Bruderer & Liechti, 1998; Diehl et al., 2003; Eastwood, 1967; Lack, 1960, 1963; Myres, 1964). Instead, our data support the observation of passerine migration to be generally more pronounced near the coastline than further offshore in the area of the German Bight (Hüppop et al., 2006, 2010). In our study, similar to the other mentioned studies, this effect is, however, partly due to a higher likelihood of observation close to the coast because our receivers were not homogeneously distributed in the study area. Our receiver array had a clear focus along the coastline (Figure 2a), which certainly adds some spatial bias to the detection data. This bias should, however, influence the detection probability of individuals of both study species in the same way. Cautious conclusions on routing behavior at species level should therefore be still valid. In both species, offshore flights occurred regularly which is in line with previous studies (e.g., Dierschke et al., 2011; Hüppop et al., 2006, 2016). We did not find species differences in the respective proportions of offshore flights. This result implies that the preferred route taken seemed to be only partly dependent on species and/or daily timing of flights. More important factors influencing routing decisions might rather comprise the conditions experienced en route (e.g., Alerstam, 1976; Brust et al., 2019; Richardson, 1990) and individual state differences (e.g., Eikenaar, Isaksson, & Hegemann, 2018; Nilsson, Brönmark, Hansson, & Chapman, 2014; Schmaljohann et al., 2013).

Taken together, our radio‐telemetry study adds knowledge on the stopover and migration behavior of individual dunnocks and blackcaps in a coastal area. Despite some limitations in the design of our telemetry array, our data challenge the general hypotheses on contrasting stopover behavior of diurnal and nocturnal migrants as well as on their contrasting routing decisions with regard to guiding landscape and open water. Our findings point to other aspects like environmental as well as individual factors being of additional importance in stopover and routing decisions rather than differences in species or daily migration timing per se. The results of our radio‐telemetry study could provide a basis to better assess potential natural and anthropogenic risks the birds might encounter *en route*. In the light of the still growing offshore wind industry in particular, our study, together with the ongoing development of micro technology in animal tracking, might help to identify specific times or environmental conditions at which individual species or species groups may be especially vulnerable to anthropogenic offshore structures (Hüppop, Michalik, et al., 2019).

## CONFLICT OF INTEREST

None declared.

## AUTHOR CONTRIBUTION

**Bianca Michalik:** Data curation (lead); Formal analysis (lead); Investigation (lead); Methodology (equal); Project administration (supporting); Visualization (equal); Writing‐original draft (equal); Writing‐review & editing (lead). **Vera Brust:** Data curation (supporting); Formal analysis (supporting); Investigation (supporting); Methodology (equal); Project administration (lead); Visualization (equal); Writing‐original draft (equal); Writing‐review & editing (supporting). **Ommo Hueppop:** Conceptualization (lead); Funding acquisition (lead); Methodology (equal); Supervision (lead); Writing‐original draft (supporting); Writing‐review & editing (supporting).

## AUTHOR CONTRIBUTION

OH initially formulated the research idea to this study conducted by BM and VB. BM prepared and analyzed the data and wrote the manuscript together with VB. All three authors commented on and agreed to the final version of the manuscript.

## Data Availability

The dataset generated and analyzed during the current study is available in the Movebank Data Repository, https://doi.org/10.5441/001/1.675pd8k5 (Michalik, Brust, & Hüppop, 2020).
